# Case Report: Prolapsed Ureterocele—A Differential Diagnosis of Urethral Cists

**DOI:** 10.1155/2020/2490129

**Published:** 2020-03-18

**Authors:** Francisco Renan Doth Sales, Georgia Alexsandra Colantonio Dourado, Ana Carolina Montes Ribeiro, Humberto de Holanda Madeira Barros, David Sucupira Cristino, Antonio Augusto Guterres Castro, Francisco José Cabral Mesquita

**Affiliations:** Department of Urology, Doctor Cesár Cals General Hospital, Fortaleza, Ceará, Brazil

## Abstract

Ureterocele is a cystic dilatation of submucosal distal ureter. It presents a higher incidence in infants and young children but is rare in adults. The urethral prolapse of ureterocele is extremely rare, and its clinical presentation includes vulvar mass, hematuria, and urinary tract dysfunction. We present a case of ureterocele prolapse in a 45-year-old woman who has a 3-day-evolution vulvar mass and intense urethral bleeding. The patient underwent armed cystoscopy and ureteroscopy, ureterocele resection, and biopsy. She evolved with good postoperative condition and was then discharged.

## 1. Introduction

Ureterocele is a cystic dilatation of submucosal distal ureter. This condition presents an incidence about 1/5000 to 1/12000 [1] and is higher in infants and young children and rare in adults and adolescents [2]. It is more frequent in women [1], and the presentation as urethral prolapse is extremely rare, appearing in only 5% of cases. [3] There are few cases in the literature presenting ureterocele prolapsed through the urethra, most of them in adult Caucasian women [3]. The diagnosis should be considered in young children presenting urinary tract infection in the first months after birth, Caucasian women with vulvar mass, urinary obstruction, and other urinary tract dysfunction associated [3].

## 2. Case Report

**Figure 1:**
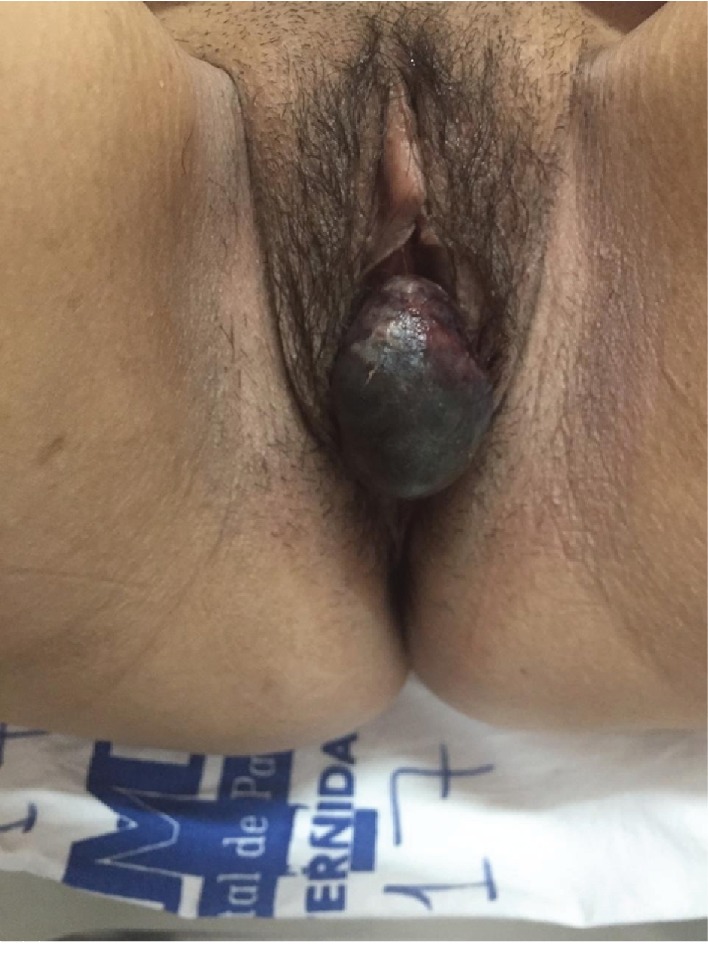
Necrotic mass in the vulvar region, requiring careful observation and examination to confirm urethral origin.

A 45-year-old patient, a menopausal woman, sought medical attention complaining of a “mass” in the genital region (Figure 1). She reported long-standing dyspareunia and a 3-day evolution of heaviness in the pelvis and the exteriorization of a “mass” in the vulvar region, associated with episodes of intense urethral bleeding. She noticed progressive change in the mucosal coloration, presented fever, and reduced urinary frequency and volume. She denied associated dysuria. Her previous pathological history includes dyslipidemia and an obstetric history of three vaginal deliveries and one miscarriage. The physical examination showed tachycardia, pallor, and a necrotic/hemorrhagic aspect of the cyst originating in the urethra, not showing correlation with the vagina. A transvaginal ultrasound was performed, showing a heterogeneous oval image, located in the vulvar region, visible on physical examination, with debris and septations in the center, measuring 4.7 × 4.0 × 3.1 cm. She underwent armed cystoscopy and ureteroscopy in which there were no injuries in the urethra and no evidence of stenosis or diverticula. It was observed an ureterocele with necrotic area and dilated right ureteral ostium and ureter. There were no anomalies in the left ureteral ostium. The exam also showed bladder with sparse trabeculations. Therefore, it was decided to perform transurethral resection of ureterocele, bleeding point hemostasis, and biopsy of the mass. The procedure occurred without complications. Patient remained on a bladder catheter for 3 days after the procedure and evolved without hematuria. The histopathological report of the surgical specimen was compatible with urothelial mucosa fragments without atypia, granulation tissue in the lamina propria, and absence of signs of neoplasia or dysplasia. The patient evolved with good postoperative condition and was then was discharged on the fifth postoperative day, after antibiotic therapy with ceftriaxone and clindamycin. After the procedure, estrogen cream was oriented.

## 3. Discussion

The first ever reported case of prolapsed ureterocele was first described in 1982 in a 45-year-old female patient with a 2-month evolution of hesitancy, nocturia, and suprapubic discomfort, in addition to a swelling in her vulvar region. [4] That report leads other medical services to consider prolapsed ureterocele as a differential diagnosis to patients who present the same findings as this first one.

The most accepted theory of the origin of ureterocele is an embryogenesis failure in the canalization of the ureteral bud [5], which would explain the predominance of the disease in infants and even more in girls than in boys (7 : 1) [6]. There are few reported cases of ureterocele in adults, and the presentation of the disease prolapsing through the urethra is even more rare [3]. Therefore, once presenting urinary complaints, a female middle-aged patient should exclude other differential diagnosis before confirming prolapsed ureterocele.

As many reports showed, including ours, the symptoms have a quick evolution, from days to months [1–4, 7–9], and are summed up on urinary symptoms (poor urinary stream, dysuria, infection, and bleeding) and the feeling or the appearance of a mass through the urethra [8]. Considering that, one of the first pathologies to discard is urethral diverticulum. Its incidence varies to 0,6%-5% [10] and is most common in middle-aged females whom had previous obstetric trauma or frequent urethral infection [6]. Its symptoms include lower urinary tract disorder, dyspareunia, post-micturition dribbling and purulent urethral discharge (this last one being pathognomonic of urethral diverticulum, which can help us to distinguish the pathologies) [6, 11]. The diagnosis is basically considering the history and performing cystourethroscopy and urethrography [11].

Another differential diagnosis is pelvic organ prolapses (POP), which is a “herniated vagina” containing one pelvic organ (bladder, rectum, or uterus) [12]. Although the urinary symptoms in the POPs are similar to the prolapsed ureterocele, such as sensation of heaviness in the pelvis, urinary urgency or frequency, voiding dysfunction, and others [13], we can distinguish from prolapsed ureterocele observing the prolapse from the vagina, not from the urethra, through pelvic examination with a Sims' speculum or a bivalve speculum [13].

## Supplementary Materials

Supplementary Materials Description of cystoscopy: visualization of the urethra and left ureteral ostium of physiological aspect. Image after transurethral resection of the right ureterocele, where we see irregular margins with edema, in addition to an enlarged right ureter.

## References

[1] Robson W. L. M., Thomason M. A., Newell R. W., Abrams R. S., Gauderer M. W. L. (1997). Picture of the month. *Archives of Pediatrics & Adolescent Medicine*.

[2] Pike S. C., Cain M. P., Rink R. C. (2001). Ureterocele prolapse—rare presentation in an adolescent girl. *Urology*.

[3] Fernández M. L., Fernández J. U., Madrid F. M. (2017). Ureterocele prolapsado: un diagnóstico para tener en cuenta ante una masa vulvar en una lactante. A propósito de un caso. *Archivos Argentinos de Pediatría*.

[4] Moore T. (1982). Orthotopic ureterocele presenting as swelling at the external urinary meatus. *British Journal of Urology*.

[5] Tanagho E. A. (1976). Embryologic basis for lower ureteral anomalies: a hypothesis. *Urology*.

[6] SMITH (2010). *Urologia Geral*.

[7] Sahu L. K., Mohanty R. (1987). Prolapsed ureterocele presenting as a vulval mass in a woman. *The Journal of Urology*.

[8] Anveden-Hertzberg L., Gauderer M. W. L., Elder J. S. (1995). Urethral prolapse: an often misdiagnosed cause of urogenital bleeding in girls. *Pediatric Emergency Care*.

[9] Miller M. A. W., Cornaby A. J., Nathan M. S., Pope A., Morgan R. J. (1994). Prolapsed ureterocele: a rare vulval mass. *British Journal of Urology*.

[10] Andersen M. J. F. (1967). The incidence of diverticula in the female urethra. *The Journal of Urology*.

[11] Davis B. L., Robinson D. G. (1970). Diverticula of the female urethra: assay of 120 cases. *The Journal of Urology*.

[12] Horst W., do Valle J. B., Silva J. C., Gascho C. L. L. (2017). Pelvic organ prolapse: prevalence and risk factors in a Brazilian population. *International Urogynecology Journal*.

[13] Jelovsek J. E., Maher C., Barber M. D. (2007). Pelvic organ prolapse. *The Lancet*.

